# Comparative outcomes of SCOM/SCOLA vs. IPOM PLUS in Umbilical and para-umbilical hernia repairs

**DOI:** 10.3389/fsurg.2026.1783033

**Published:** 2026-05-29

**Authors:** Kumaresh Pandian, Nehar Sai Surya Srikanth Saride, Naveen Alexander, K. Arun Kumar

**Affiliations:** Department of General Surgery, Sri Ramachandra Institute of Higher Education and Research, Chennai, India

**Keywords:** cost-effectiveness, hospital stay, IPOM plus, laparoscopic hernia repair, para-umbilical hernia, postoperative pain, SCOM/SCOLA, seroma

## Abstract

**Background:**

Umbilical and para-umbilical hernias are common abdominal wall defects, often requiring surgical intervention to prevent complications. The Subcutaneous Onlay Laparoscopic Approach (SCOM/SCOLA) and Laparoscopic Intraperitoneal Onlay Repair Plus (IPOM PLUS) are two laparoscopic techniques employed for hernia repair. This study aims to compare these two methods based on surgical time, postoperative pain, seroma formation, hospital stay, and overall cost to guide the selection of appropriate treatment strategies.

**Methods:**

This prospective observational study included 60 adult patients with umbilical or para-umbilical hernias treated at a tertiary care hospital between February 2022 and December 2022. Patients were divided equally into two groups, with 30 undergoing IPOM PLUS and 30 receiving SCOM/SCOLA. Surgical outcomes were evaluated based on duration of surgery, postoperative pain scores, seroma formation during the first and third weeks, hospital stay length, and overall cost of care. Statistical comparisons were performed using appropriate tests with *p* < 0.05 considered significant.

**Results:**

IPOM PLUS demonstrated a significantly shorter mean surgical time (79.13 ± 12.73 min) compared to SCOM/SCOLA (94.53 ± 21.25 min, *p* = 0.001). Postoperative pain relief was superior in the SCOM/SCOLA group, with significantly lower pain levels observed from the 7th day onward (*p* < 0.0001). The incidence of seroma was higher in the SCOM/SCOLA group (86.7% in the 1st week) compared to IPOM PLUS (6.7%). By the third week, seroma rates declined to 20% and 3.3% in the SCOM/SCOLA and IPOM PLUS groups, respectively. The IPOM PLUS group had shorter hospital stays (1.83 ± 0.79 days) than the SCOM/SCOLA group (5.07 ± 1.36 days, *p* < 0.0001). Although IPOM PLUS had higher procedural costs, SCOM/SCOLA incurred greater total costs due to prolonged hospitalization.

**Conclusion:**

Both IPOM PLUS and SCOM/SCOLA are effective surgical techniques with distinct advantages. IPOM PLUS provides faster surgical times, shorter hospital stays, and fewer complications, making it ideal for patients needing rapid recovery. In contrast, SCOM/SCOLA offers better postoperative pain relief and cost-effectiveness per procedure but carries a higher risk of seroma and longer hospital stays. Tailoring the choice of surgical technique to individual patient needs and clinical circumstances can optimize outcomes and resource utilization.

## Introduction

Umbilical and para-umbilical hernias represent a significant proportion of primary ventral hernias encountered in abdominal wall surgery. Despite advances in surgical technique, the optimal approach remains controversial, particularly with regard to balancing operative complexity, postoperative pain, complications, and recurrence. In recent years, the management of ventral hernias has increasingly shifted toward minimally invasive surgical techniques, which aim to reduce wound complications, postoperative pain, and hospital stay. Several laparoscopic and robotic approaches have been described, including intraperitoneal onlay mesh repair (IPOM), enhanced-view totally extraperitoneal repair (eTEP), transabdominal preperitoneal repair (TAPP), and robotic-assisted retromuscular repairs (1). These techniques differ primarily in the plane of mesh placement, which significantly influences postoperative outcomes and complication profiles.

Laparoscopic intraperitoneal onlay mesh (IPOM) repair has long been a mainstay for ventral hernia management, offering advantages in terms of reduced wound morbidity and shorter hospital stays compared to open techniques (2–4). However, concerns remain about mesh-viscera interface complications, seroma formation, and chronic pain when defect closure is omitted (i.e., bridging). To mitigate these, IPOM PLUS variants—which include primary defect closure before mesh placement—have been introduced, with promising early results in reduced mesh bulging, lower seroma rates, and improved abdominal wall function (5–7).

The Subcutaneous Onlay Mesh (SCOM) repair, also referred to as Subcutaneous Onlay Laparoscopic Approach (SCOLA) in some reports, involves laparoscopic dissection of the subcutaneous space with placement of a mesh in the onlay position above the anterior rectus sheath. This technique may avoid the risks of intra-abdominal adhesion or mesh–viscera complications, while still providing reinforcement of the defect (8). Early comparative studies suggest that SCOLA may confer less postoperative pain, especially in selected patients without redundant skin, though concerns about seroma and long-term durability remain (9).

SCOM/SCOLA may be particularly considered in selected patients with small primary midline ventral hernias, especially when avoidance of intraperitoneal mesh is preferred or when access to advanced techniques such as robotic surgery or eTEP is limited. Despite increasing interest in this approach, comparative data evaluating SCOM/SCOLA against established laparoscopic techniques such as IPOM PLUS remain limited, particularly in real-world clinical settings.

Therefore, the present study aimed to compare the short-term surgical outcomes of IPOM PLUS and SCOM/SCOLA techniques in the management of umbilical and para-umbilical hernias, focusing on operative time, postoperative pain, seroma formation, hospital stay, and overall cost of treatment.

## Aim of the study

In accordance with principles advocated by STROBE for transparent observational reporting, the aim of this study is to compare IPOM PLUS vs. SCOM/SCOLA techniques in the repair of umbilical and para-umbilical hernia, evaluating operative time, postoperative complications (including seroma), pain outcomes, hospital stay, and recurrence.

## Methods

Researchers conducted a prospective observational study between February 2022 and December 2022 at the Department of General Surgery, Sri Ramachandra Institute of Higher Education and Research. 60 patients were enrolled for the study. Patients were allocated into two groups in an alternate sequence, such that one patient underwent SCOM/SCOLA and the next underwent IPOM PLUS. This ensured equal distribution of cases between the two groups (systemic allocation). However this does not eliminate the risk of selection bias as would true randomization.

The relatively small sample size reduces the statistical power may limit the generalizability of our results. However, all procedures were performed by a single surgeon with optimum skills in advanced laparoscopic surgery for over 5 years, in a single operation theatre complex under general anaesthesia. This ensured technical consistency and minimized variability attributable to surgeon experience or operative setting.

To ensure uniformity, mesh material was standardized across patients in each group. In the IPOM PLUS group, BARD ECHO-PS mesh [lightweight, composite, dual-sided mesh with an absorbable barrier] was used, while in the SCOM/SCOLA group, a lightweight macro porous polypropylene mesh (15 × 15 cm) was employed. The same mesh type and weight were used consistently for all patients within each arm of the study. In the IPOM PLUS group, mesh was fixed using with absorbable tacks. The primary fascial defect closure was performed intracorporeally using 2–0 V-Loc sutures ensuring complete approximation of the defect edges before mesh placement.

In the SCOM/SCOLA group, mesh was secured in the subcutaneous plane with interrupted sutures. The fixation technique was standardized for all patients within each arm. In the SCOM/SCOLA group, subcutaneous drains were routinely placed and removed once the drainage had reduced to minimal levels, typically after 7–8 days. No routine drains were used in the IPOM PLUS group. In this study, SCOM/SCOLA was selectively used for small primary midline hernias (≤5 cm), non-obese patients, absence of redundant skin, and when avoidance of intraperitoneal mesh was preferred, including patient-specific or surgeon-driven considerations.

This study included adult patients (≥18 years) presenting with a single, primary midline ventral hernia with a defect size of 5 cm or less. According to the European Hernia Society (EHS) classification, eligible hernias comprised umbilical hernias (defects within 3 cm of the umbilicus) and adjacent midline hernias such as small epigastric (above the umbilicus) or infra umbilical (below the umbilicus) hernias. Hernias lateral to the linea alba (e.g., Spigelian, lumbar) were excluded, as were multiple or recurrent hernias, incisional hernias, and emergency presentations (obstructed or strangulated hernias). The primary outcome of the study assessed the postoperative pain measured by Visual Analogue Scale. The secondary outcomes of the study included operative time, incidence of seroma (at 1st and 3rd weeks), duration of hospital stay, procedural and total hospitalization costs.

Postoperative pain was assessed using the Visual Analogue Scale (VAS), a 10-point scale ranging from 0 (no pain) to 10 (worst imaginable pain). Pain scores were recorded at predefined time points: postoperative day 1, day 3, day 7, and at weeks 3 and 6 during follow-up visits. All assessments were performed during routine clinical evaluation by the surgical team. All patients received a standardized postoperative analgesic regimen according to institutional protocols. Intravenous paracetamol (1 g every 8 h) was administered during the immediate postoperative period, with non-steroidal anti-inflammatory drugs (NSAIDs) provided as required for additional pain control. Once patients resumed oral intake, analgesia was transitioned to oral paracetamol and NSAIDs as needed. The same analgesic protocol was followed for both groups to ensure consistency in pain management and comparability of pain scores.

Seroma was defined as a clinically detectable postoperative fluid collection at the operative site without signs of infection. Diagnosis was based primarily on physical examination (visible swelling or palpable fluctuant collection). Ultrasonography was performed when clinical findings were equivocal or when quantification was required.

### Surgical technique

#### IPOM PLUS technique

Patients were positioned in the supine position, and pneumoperitoneum was established. A 30° laparoscope was introduced to access the abdominal cavity and visualize the hernia defect. After a thorough 360° inspection of the peritoneal cavity, adhesiolysis was performed as required to release any adhesions between the abdominal wall and intra-abdominal contents. The hernia contents were then reduced into the abdominal cavity. Surrounding structures that could interfere with adequate mesh placement, including the peritoneum as well as the umbilical and falciform ligaments, were carefully dissected when necessary.

**Figure 1:**
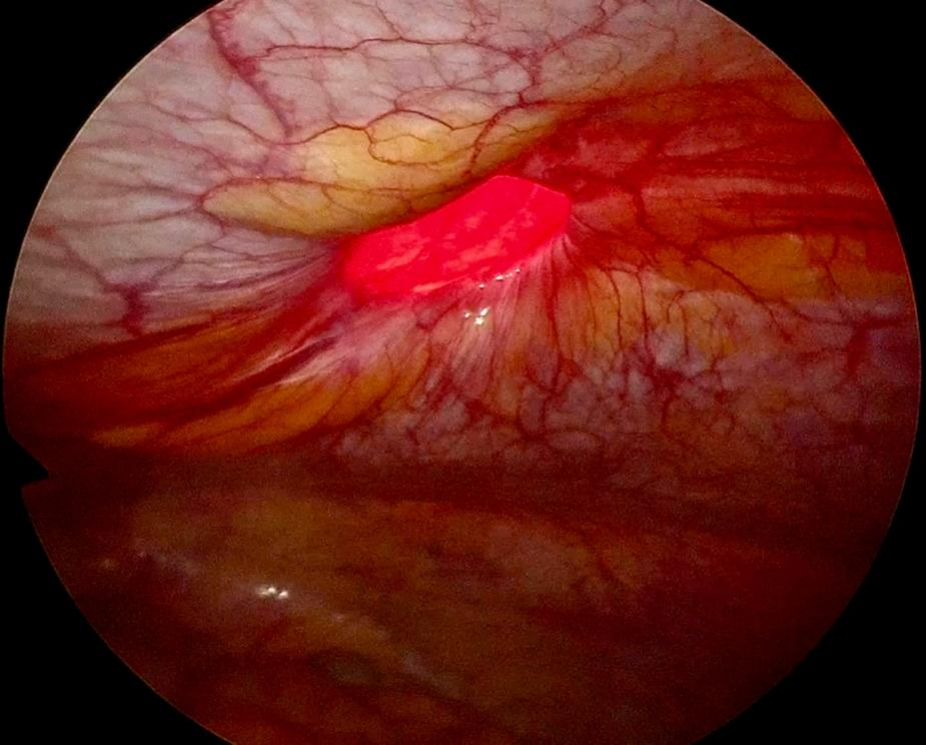
Hernial defect.

**Figure 2:**
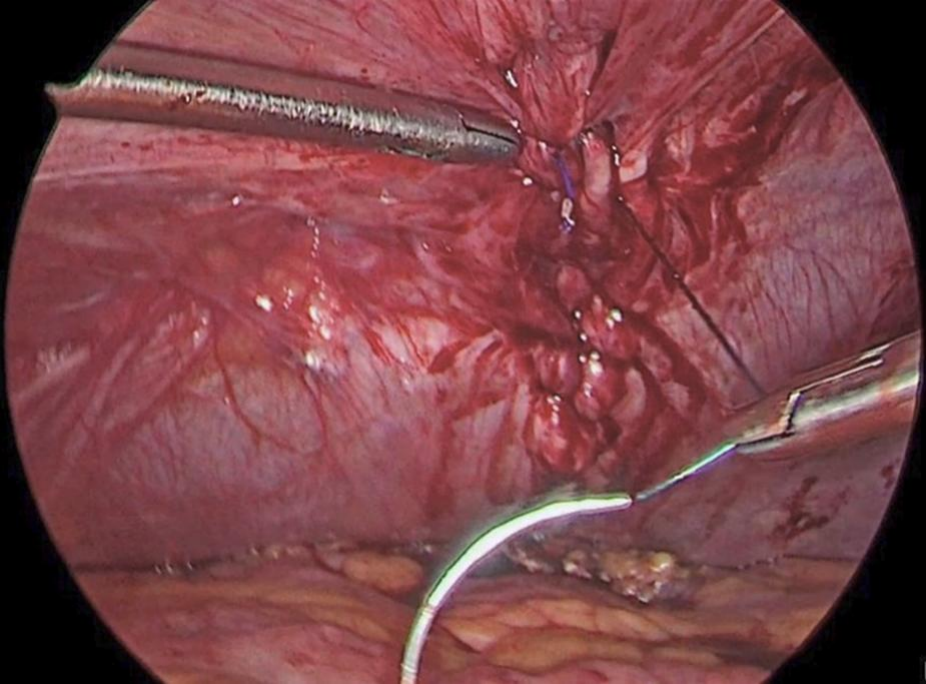
Defect closure with barbed suture.

**Figure 3:**
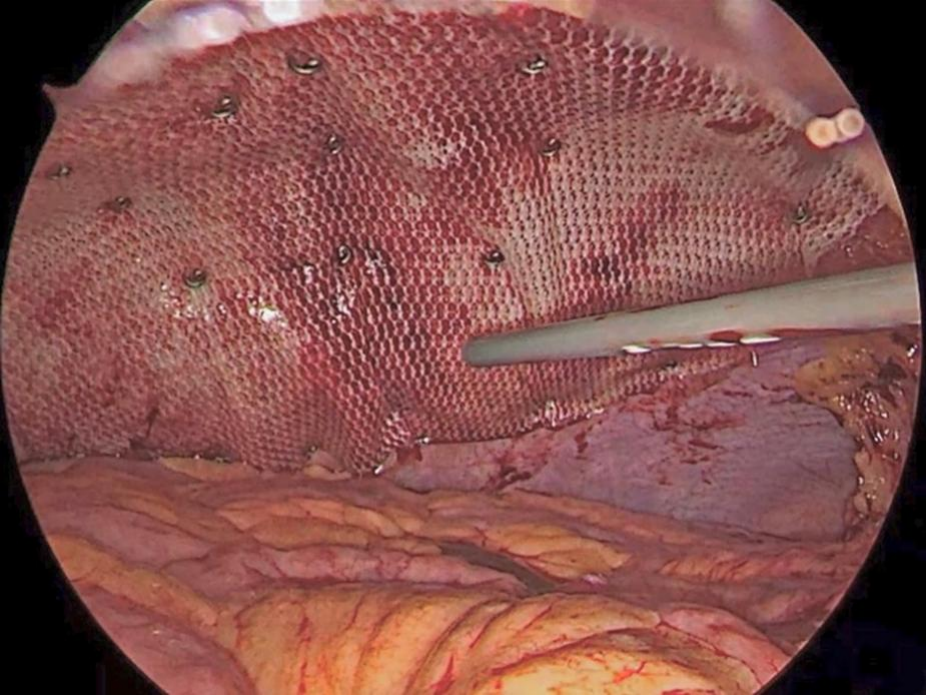
Mesh fixation using tackers.

The fascial defect was measured under direct laparoscopic visualization. Primary closure of the defect was performed using non-absorbable barbed sutures, constituting the “plus” component of the procedure. Subsequently, a composite mesh was deployed intraperitoneally and secured using absorbable tacks in a double-crown fixation technique, ensuring a minimum mesh overlap of 5 cm beyond the defect margins to reduce the risk of recurrence (10) (Figures 1–3).

#### SCOM/SCOLA technique

The patient was placed in the supine position under general anaesthesia. A working space was created in the subcutaneous plane above the anterior rectus sheath using blunt and sharp dissection with carbon dioxide insufflation to maintain the operative field. Laparoscopic ports were inserted in the subcutaneous space, and a 30° laparoscope was used for visualization.

Dissection was continued to develop the subcutaneous plane around the hernia defect, allowing adequate exposure of the anterior abdominal wall fascia. The hernia sac contents were reduced, and the fascial defect was identified and measured under direct visualization. Primary closure of the defect was performed using non-absorbable barbed sutures.

**Figure 4:**
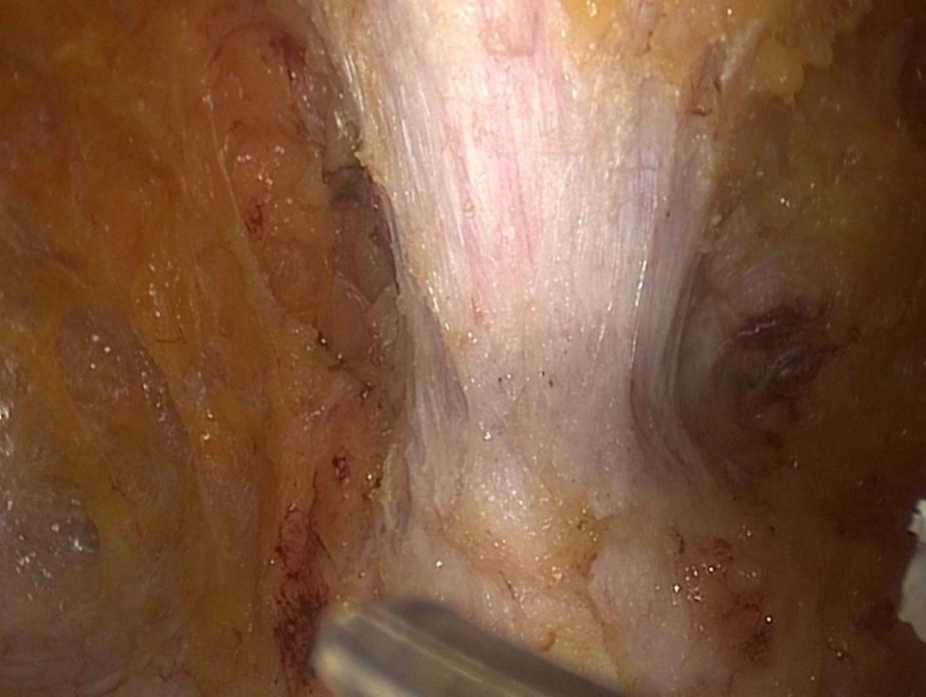
Subcutaneous plane creation along with hernial sac.

**Figure 5:**
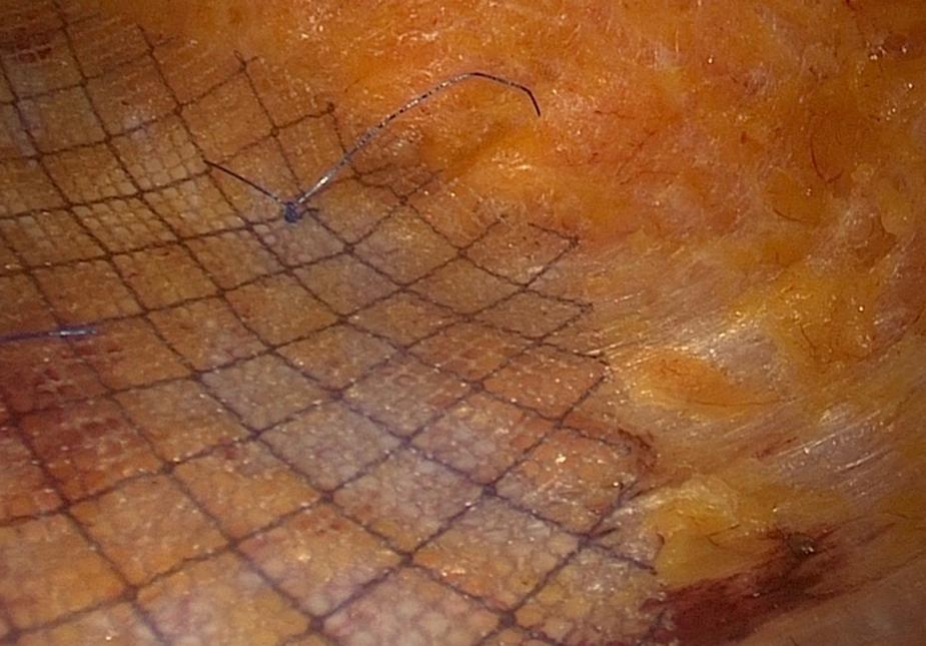
Mesh fixation over the anterior rectus sheath.

**Figure 6:**
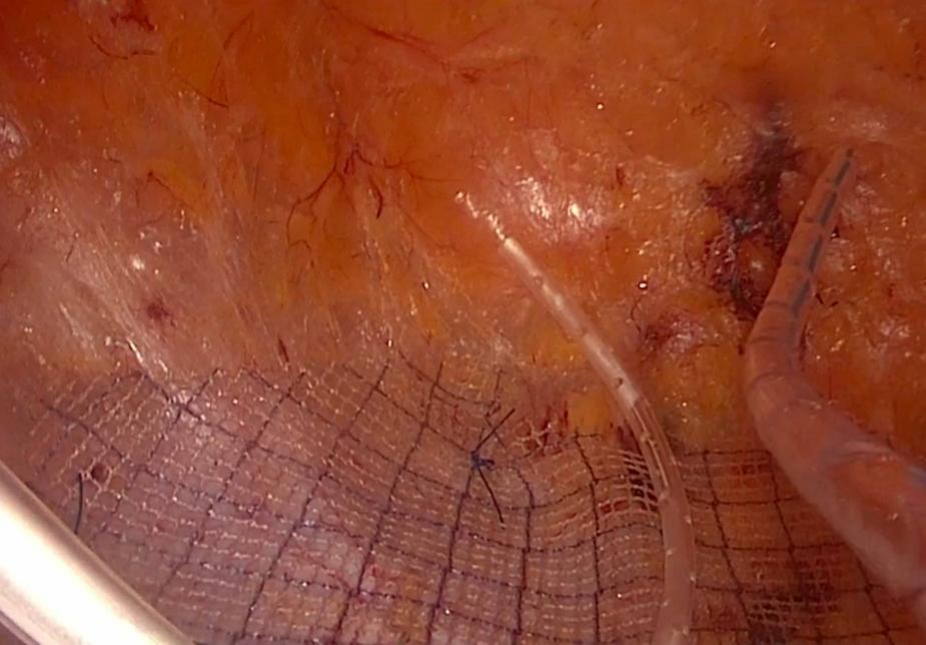
Subcutaneous drain placement.

A lightweight polypropylene mesh was then placed in the onlay position over the anterior rectus sheath, ensuring adequate overlap of at least 5 cm beyond the margins of the defect. The mesh was secured using interrupted sutures. A closed suction drain was placed in the subcutaneous space to prevent fluid accumulation and was removed once drainage was minimal. (Figures 4–6)

#### Postoperative follow-up

All patients were followed postoperatively at 1 week, 4 weeks, and 6 weeks after surgery. Additional visits were arranged if patients reported symptoms or complications. During each follow-up visit, patients underwent a clinical examination to evaluate wound healing, seroma formation, hematoma, surgical site infection, and early recurrence.

#### Assessment of postoperative complications

Postoperative complications including seroma, hematoma, and surgical site infection (SSI) were assessed through clinical examination during follow-up visits.

**Figure 7:**
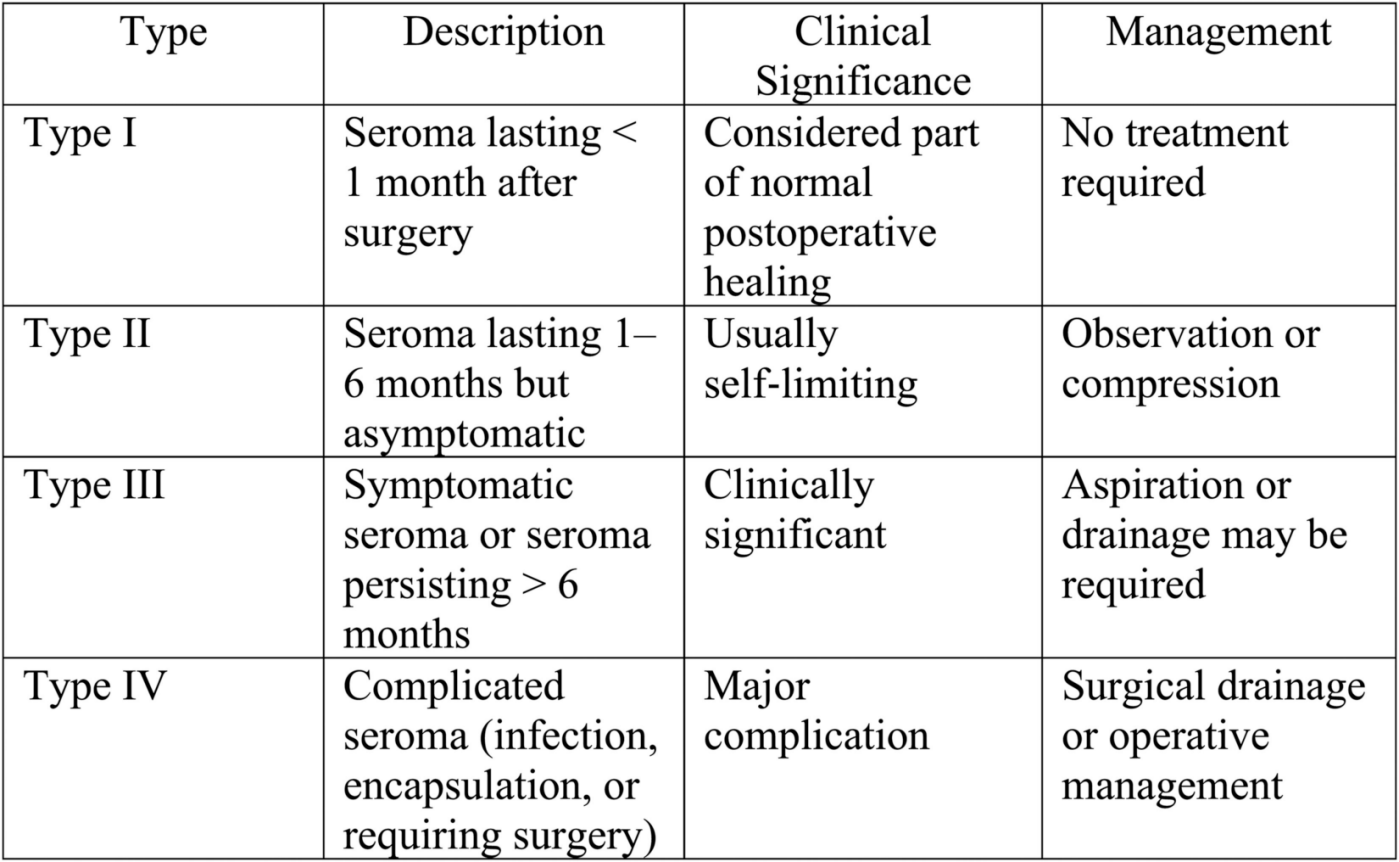
Classification of postoperative seroma following ventral hernia repair according to the international endohernia society (IEHS).

- Seroma was defined as a clinically detectable fluid collection at the operative site, confirmed by ultrasound when necessary, and classified according to the International Endohernia Society (IEHS) classification (Figure 7).
- Hematoma was defined as a localized collection of blood within the surgical area presenting with swelling, discoloration, or confirmed by imaging.
- Surgical site infection was diagnosed according to the criteria of the Centres for Disease Control and Prevention, including localized redness, warmth, swelling, purulent discharge, or positive wound culture.

#### Management of complications

Postoperative complications were managed according to standard clinical protocols.

- Seromas were managed conservatively with observation and compression. Symptomatic or persistent collections were treated with percutaneous aspiration under sterile conditions.
- Hematomas were managed conservatively unless large or symptomatic, in which case evacuation was performed.
- Surgical site infections were treated with appropriate antibiotic therapy, wound care, and drainage if required.

#### Postoperative discharge criteria

Patients were discharged once they were hemodynamically stable, tolerating oral intake, ambulatory, and free of complications requiring inpatient monitoring. In the SCOM/SCOLA group, discharge was additionally dependent on subcutaneous drain management, including monitoring of drain output and removal once output reached minimal levels. Pain severity alone was not used as a criterion for continued hospitalization.

## Results

### Age distribution

**Figure 8:**
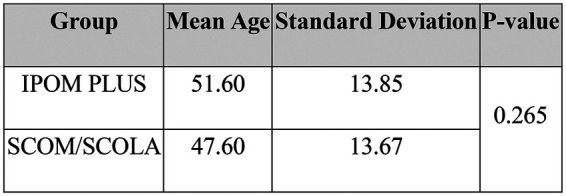
Age distribution of patients.

The study included 60 patients divided evenly between the IPOM PLUS and SCOM/SCOLA groups. The mean age of participants in the IPOM PLUS group was 51.60 ± 13.85 years, while the SCOM/SCOLA group had a mean age of 47.60 ± 13.67 years (Figure 8). The difference in age between the two groups was not statistically significant (*p* = 0.265).

### Gender distribution

**Figure 9:**
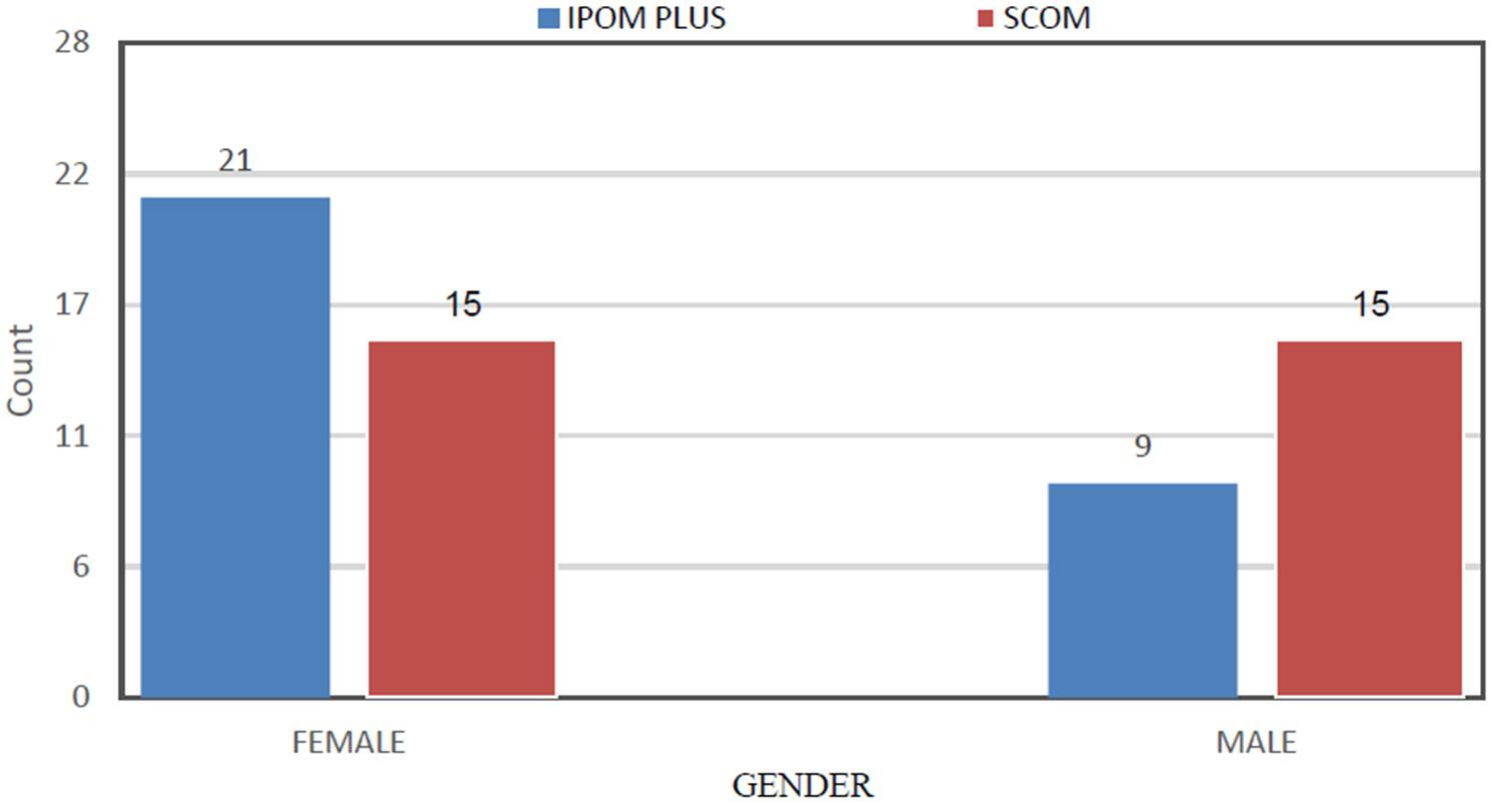
Gender distribution of the patients.

The gender distribution varied slightly between the two groups. The IPOM PLUS group had a female predominance, with 21 females and 9 males. In contrast, the SCOM/SCOLA group had an equal number of male and female participants, with 15 each (Figure 9). Overall, more women participated in the study.

### BMI comparison

**Figure 10:**
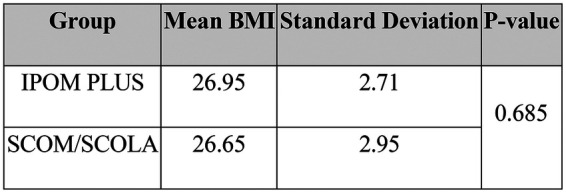
BMI in patients.

Both groups showed similar body mass index (BMI) values. The mean BMI for the IPOM PLUS group was 26.95 ± 2.71, while the SCOM/SCOLA group reported a mean BMI of 26.65 ± 2.95 (Figure 10). This similarity was confirmed with a *p*-value of 0.685, indicating no significant difference between the groups.

### Hernia diagnosis

**Figure 11:**
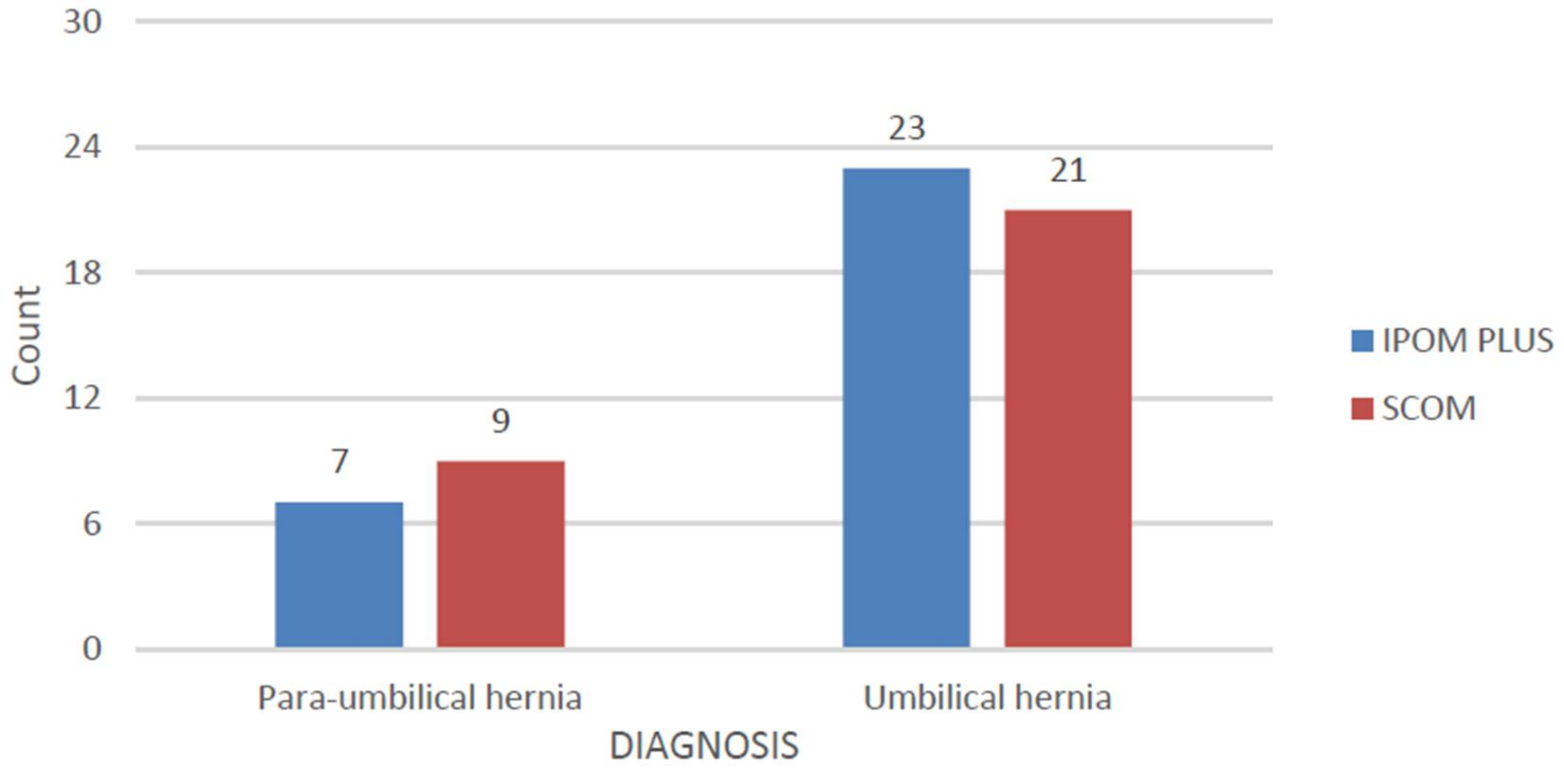
Diagnosis of the patients.

The diagnoses of the patients included either para-umbilical or umbilical hernias. The IPOM PLUS group had 23 patients (76.7%) with umbilical hernias and 7 patients (23.3%) with para-umbilical hernias. In the SCOM/SCOLA group, 21 patients (70%) had umbilical hernias, and 9 patients (30%) had para-umbilical hernias (Figure 11).

### Defect size

**Figure 12:**
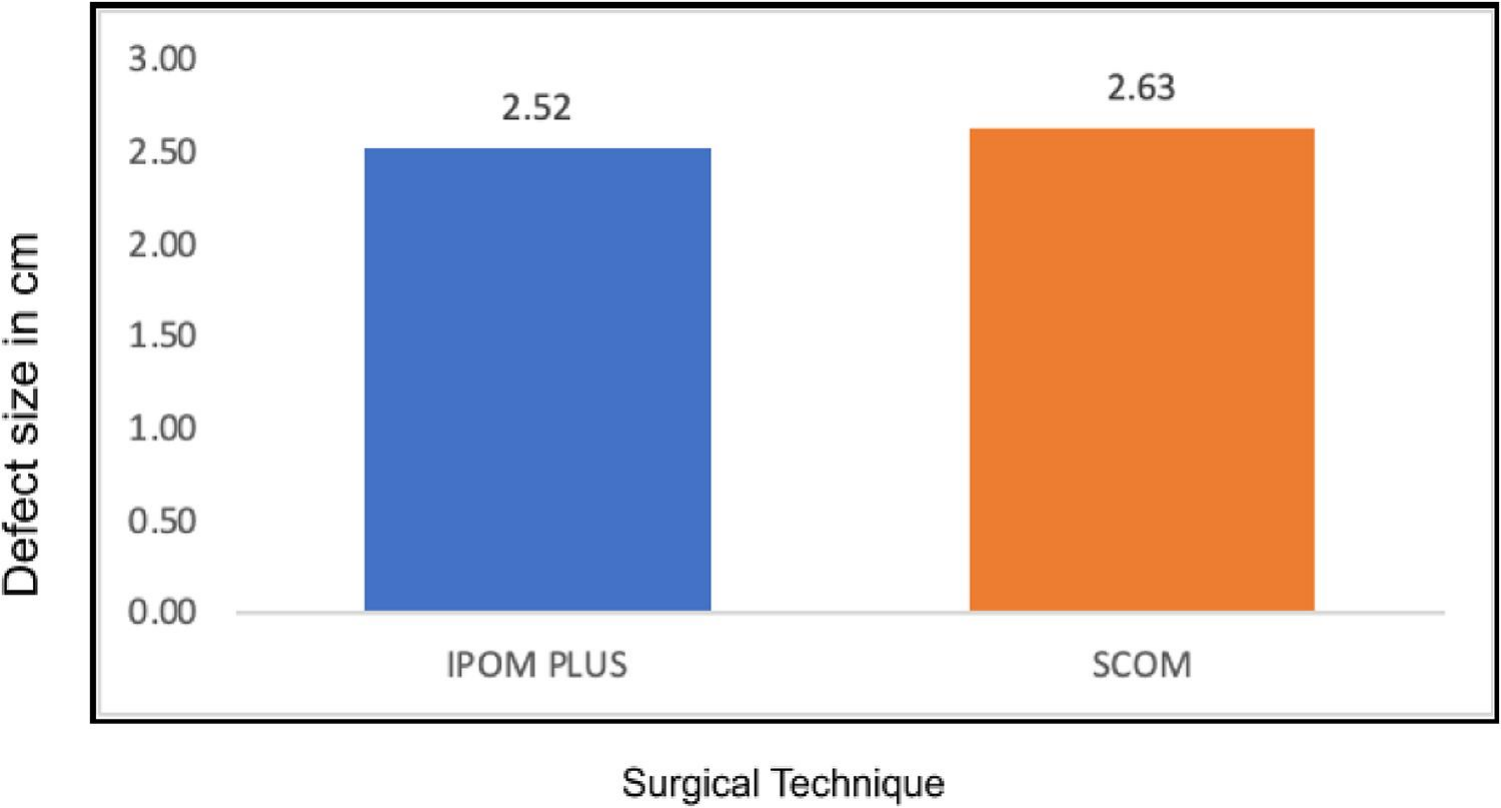
Distribution of defect size in patients and laparoscopic methods.

The mean defect size among participants was comparable between the two groups. The IPOM PLUS group reported a mean defect size of 2.52 ± 1.20 cm, and the SCOM/SCOLA group had a mean defect size of 2.63 ± 1.38 cm (Figure 12). There was no statistically significant difference in defect sizes between the two groups (*p* = 0.783).

### Surgical duration

**Figure 13:**
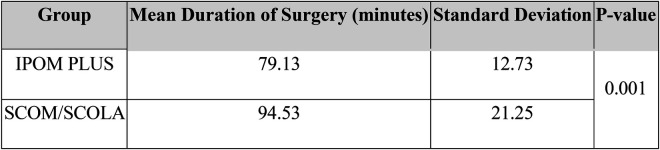
Surgical hours in patients.

The IPOM PLUS technique resulted in shorter surgical times compared to the SCOM/SCOLA approach. The mean duration for IPOM PLUS was 79.13 ± 12.73 min, while the SCOM/SCOLA group required 94.53 ± 21.25 min (Figure 13). The difference was statistically significant, with a *p*-value of 0.001.

### Seroma formation

**Figure 14:**
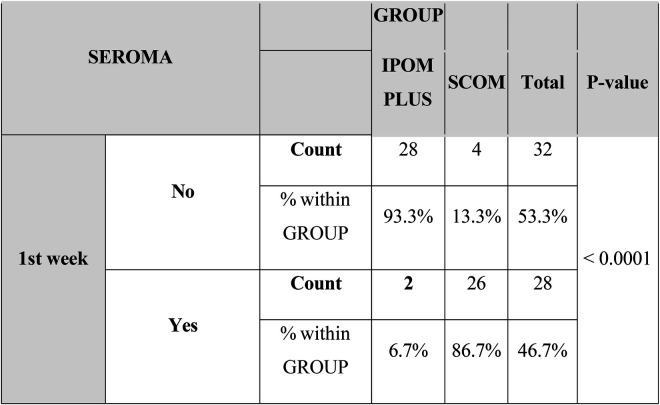
Seroma in participants.

**Figure 15:**
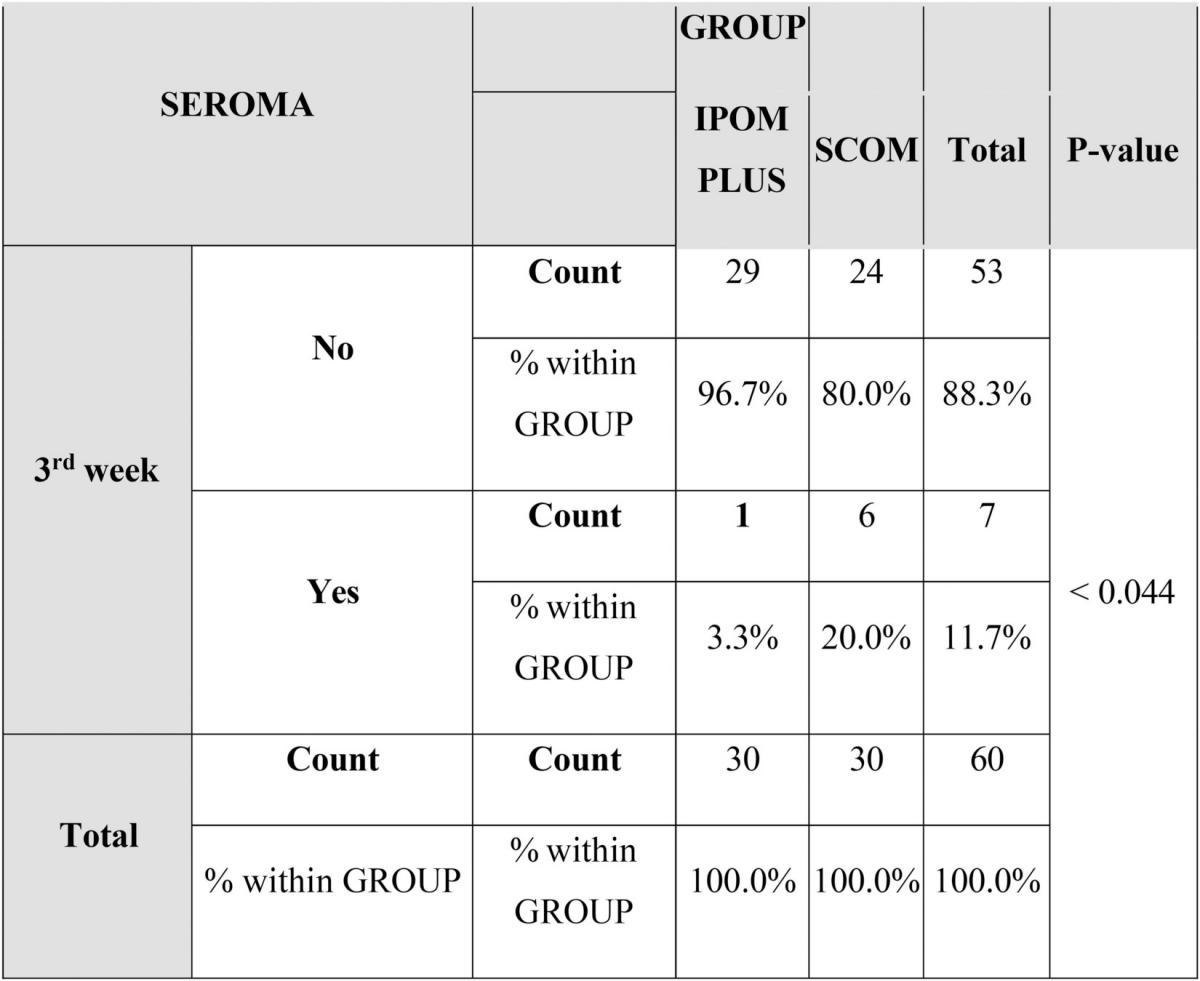
3rd-week follow-up for seroma.

The development of seroma was significantly different between the two groups. In the first week after surgery, 26 patients (86.7%) in the SCOM/SCOLA group developed seroma, compared to only 2 patients (6.7%) in the IPOM PLUS group (Figure 14). By the third week, the incidence of seroma decreased to 6 patients (20%) in the SCOM/SCOLA group and 1 patient (3.3%) in the IPOM PLUS group (Figure 15). All seromas observed in this study were Type 1 or Type 2 and were managed conservatively without invasive intervention. All early postoperative seromas resolved spontaneously with conservative management, and no persistent seroma was observed at 6 weeks.

**Figure 16:**
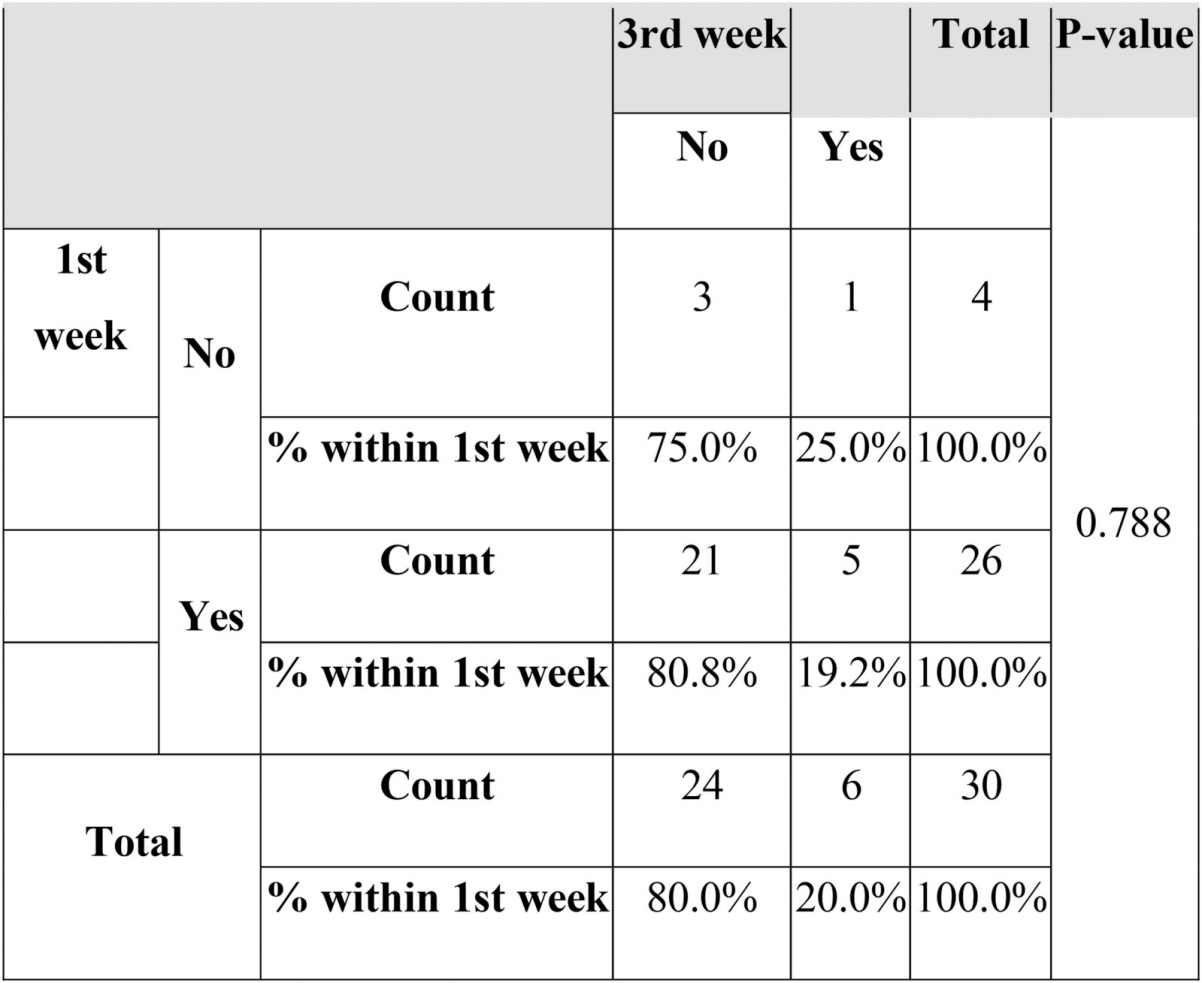
Comparison of 1st and 3rd-week seroma follow-up in the SCOM group.

The comparison of seroma formation within the IPOM PLUS group between the first and third weeks showed no significant difference (Figure 16). Two patients had seroma in the first week, but only one patient continued to show seroma by the third week.

In the SCOM/SCOLA group, 26 patients developed seroma in the first week, but only 6 patients had seroma by the third week. The reduction in seroma formation between the two follow-ups did not reach statistical significance. At the 6-week follow-up, no patients in either the IPOM PLUS or SCOM/SCOLA group had persistent seroma.

### Postoperative pain assessment

**Figure 17:**
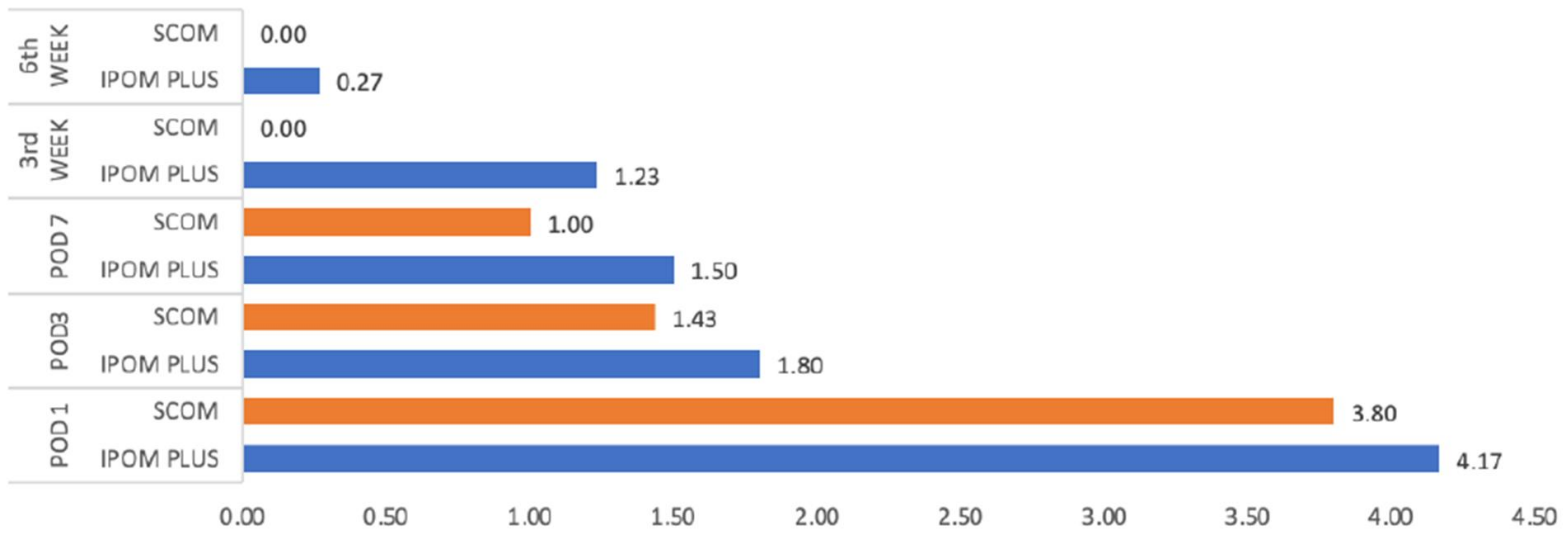
Comparison of IPOM PLUS and SCOM group for weekly follow-up.

Postoperative pain scores were measured on the 1st, 3rd, and 7th day, and at the 3rd and 6th week. The SCOM/SCOLA group reported significantly lower pain levels compared to the IPOM PLUS group. By the 7th day, pain levels decreased markedly in the SCOM/SCOLA group, and by the 3rd and 6th weeks, no patients in this group reported pain. In contrast, the IPOM PLUS group showed higher pain scores initially, though pain subsided by the 6th week (Figure 17).

### Hospital stay duration

**Figure 18:**
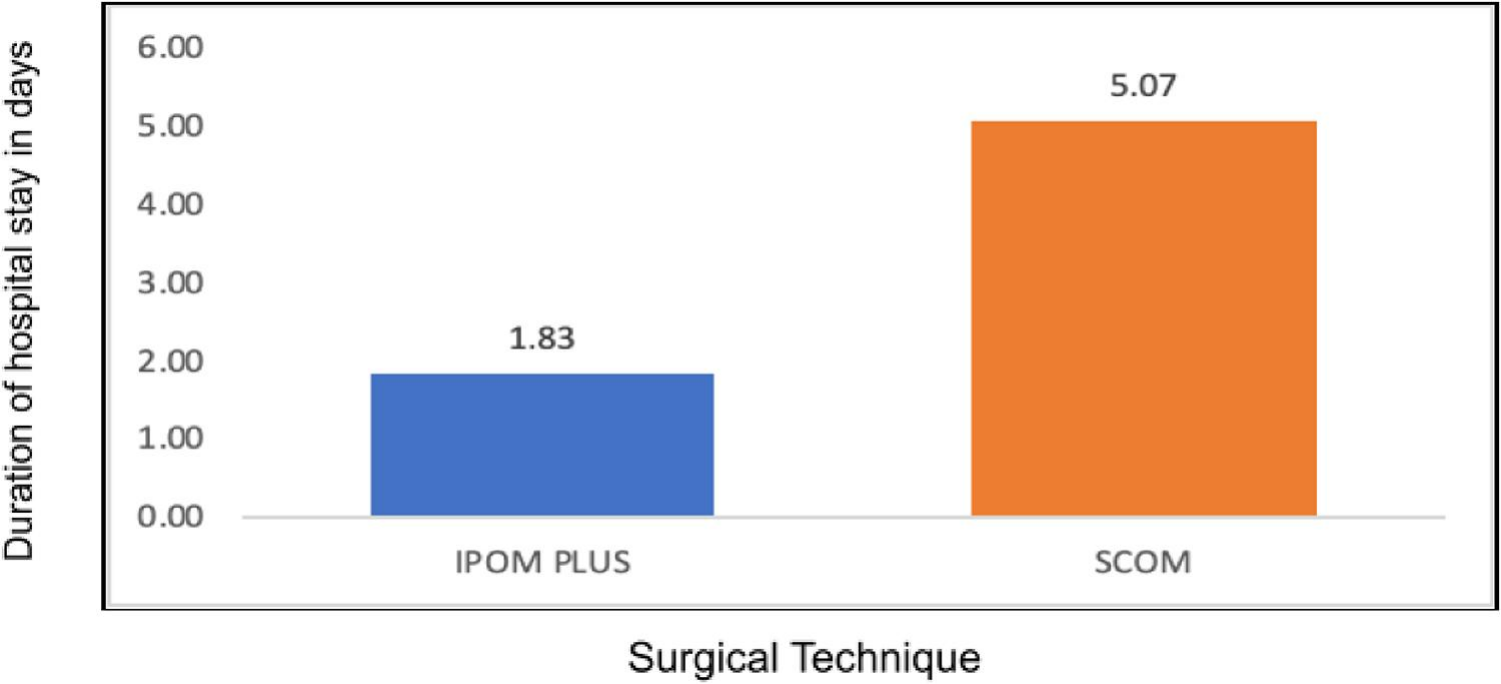
Duration of hospital stay in patients.

The duration of hospital stay was significantly shorter for the IPOM PLUS group compared to the SCOM/SCOLA group. Patients in the IPOM PLUS group had an average stay of 1.83 ± 0.79 days, while those in the SCOM/SCOLA group stayed for 5.07 ± 1.36 days (Figure 18). The difference between the two groups was statistically significant (*p* < 0.0001). Patients undergoing SCOM/SCOLA had routine placement of subcutaneous drains. Hospital stay was prolonged primarily due to drain output monitoring, need for daily assessment of drain volume, and risk of early postoperative seroma accumulation in the subcutaneous dead space. Drains were removed only once output reduced to minimal levels, which typically occurred between postoperative days 5–6 in our institutional protocol.

### Cost of surgery

**Figure 19:**
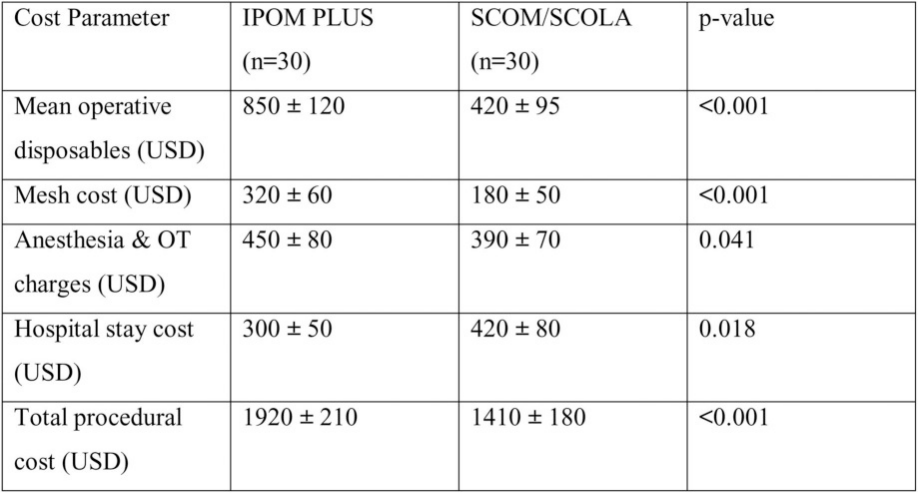
Cost of surgery.

The mean total procedural cost was significantly higher in the IPOM PLUS group compared with the SCOM/SCOLA group (1,920 ± 210 vs. 1,410 ± 180 USD, *p* < 0.001) (Figure 19). The higher cost in the IPOM PLUS group was largely attributable to more expensive operative disposables and mesh, despite shorter hospital stays reducing ward costs. These findings indicate that IPOM PLUS entails a higher upfront procedural expense compared to SCOM/SCOLA.

## Discussion

In recent years, there has been increasing interest in extraperitoneal and preperitoneal approaches for ventral hernia repair, including enhanced-view totally extraperitoneal repair (eTEP) and transabdominal preperitoneal repair (TAPP). These techniques aim to combine the advantages of minimally invasive surgery with placement of mesh outside the peritoneal cavity, thereby avoiding direct mesh–viscera contact while preserving the biomechanical benefits of retro muscular reinforcement. Several studies have reported favourable outcomes with these approaches, including reduced postoperative pain and improved functional recovery, although they may require greater technical expertise and longer operative times (1, 5).

This prospective observational study compared two laparoscopic approaches for umbilical and para-umbilical hernia repair: IPOM PLUS and SCOM/SCOLA. The study demonstrated that IPOM PLUS was associated with significantly shorter operative time, lower seroma formation, and shorter hospital stay, albeit with higher procedural costs and greater early postoperative pain. Conversely, SCOM/SCOLA provided superior pain relief and lower upfront procedural costs, but was associated with higher seroma rates and longer hospitalization. These findings confirm that both techniques are effective, yet each offers distinct advantages that may be tailored to individual patient needs and healthcare system priorities.

The longer hospital stay observed in the SCOM/SCOLA group should be interpreted in the context of postoperative management protocols rather than as an inherent disadvantage of the surgical technique itself. In our institution, routine placement of subcutaneous drains is standard practice following SCOM/SCOLA due to the creation of a potential dead space in the subcutaneous plane. Patients were typically monitored in hospital until drain output decreased to minimal levels and the drain could be safely removed. Consequently, the duration of hospitalization in this cohort was largely influenced by postoperative protocol rather than by differences in postoperative recovery or surgical complexity. In contrast, IPOM PLUS did not require routine drain placement, allowing earlier discharge. Therefore, the difference in hospital stay between the two groups should be interpreted primarily as a protocol-driven outcome rather than a direct technique-related effect. Future studies comparing these techniques should ideally standardize postoperative management protocols to better isolate the effect of the surgical technique itself on recovery outcomes.

The strengths of this study include its prospective design, uniform data collection, and comprehensive assessment of surgical, postoperative, and economic outcomes. Both groups were comparable in terms of baseline demographics, BMI, and defect size, which minimizes confounding. Furthermore, the inclusion of cost analysis adds practical relevance for health systems decision-making.

Limitations include the single-centre nature, relatively small sample size (*n* = 60), and short follow-up, which preclude definitive conclusions about recurrence and long-term mesh-related complications. Additionally, cost calculations were based on institutional pricing and may not reflect other healthcare environments. Subjective pain scoring introduces patient-related bias, and the absence of blinding could influence postoperative care and reporting. Although baseline variables such as age, sex, BMI, and hernia defect size were comparable between groups, multivariate adjustment for potential confounders was not performed due to the relatively small sample size. This represents a limitation, and future larger studies should incorporate multivariate analyses to better control for potential confounding.

Additionally, the cost comparison presented in this study represents a basic institutional cost analysis rather than a comprehensive cost-effectiveness evaluation. The analysis was limited to direct procedural and hospitalization costs and did not incorporate indirect costs, long-term healthcare utilization, or patient-reported outcomes. Therefore, the economic findings should be interpreted cautiously, and future studies incorporating formal cost-effectiveness methodologies are warranted.

Another factor that may influence postoperative outcomes is the type of mesh used and the method of fixation. In the present study, a composite dual-sided mesh with an absorbable barrier (BARD ECHO-PS mesh) fixed with absorbable tacks was used for IPOM PLUS, whereas a lightweight polypropylene mesh secured with sutures was used for SCOM/SCOLA. Differences in mesh material, weight, and fixation technique may affect postoperative pain, inflammatory response, and seroma formation. For example, intraperitoneal fixation using tackers or trans-fascial sutures may contribute to increased early postoperative pain, whereas the creation of a subcutaneous space in SCOM/SCOLA may predispose to fluid accumulation and seroma formation. Although mesh type and fixation were standardized within each group, their potential influence on outcomes should be considered when interpreting the results.

Our findings corroborate prior evidence suggesting that defect closure in IPOM PLUS significantly reduces seroma incidence compared to traditional IPOM bridging techniques. Chelala et al. and Bittner et al. emphasized the importance of fascial closure in preventing bulging and seroma formation, consistent with our results (11, 12). Similarly, Muysoms et al. highlighted the high seroma risk associated with onlay repairs, which parallels the findings in our SCOM/SCOLA cohort (13). Seroma formation was assessed primarily through clinical examination, with ultrasonography performed selectively when findings were equivocal. This approach may introduce detection bias, as small or asymptomatic fluid collections may be under- or overestimated. Furthermore, differences in the anatomical plane of mesh placement between the two techniques may influence clinical detection. Subcutaneous approaches such as SCOM/SCOLA create a potential dead space that can lead to more clinically detectable fluid collections, whereas intraperitoneal approaches may conceal smaller seromas that remain asymptomatic. Therefore, the relatively high early seroma rate observed in the SCOM/SCOLA group should be interpreted with caution, as many of these collections were clinically mild and resolved spontaneously with conservative management. Importantly, the majority of seromas in this study corresponded to low-grade collections and did not require invasive intervention, suggesting limited clinical impact despite the higher early incidence.

Several comparative studies have investigated the advantages and drawbacks of IPOM vs. alternative laparoscopic techniques. Köckerling et al. demonstrated that IPOM PLUS provides lower recurrence rates and fewer complications when fascial closure is performed (14). Sarli et al. and Prasad et al. found that TAPP or preperitoneal mesh placements were associated with lower postoperative pain but longer operative times compared to IPOM, which mirrors the trade-off observed in our study (15, 16). More recent studies by Megas et al. and Kumar et al. comparing eTEP and IPOM PLUS reported that eTEP offers reduced pain and improved functional outcomes but is technically more demanding and time-consuming (17, 18).

Regarding hospital stay, our findings are consistent with Leblanc et al. and Misra et al., who observed that IPOM PLUS patients experienced shorter stays compared to other laparoscopic approaches (19, 20). However, consistent with our results, onlay or subcutaneous techniques like SCOLA were associated with better postoperative pain outcomes but required more prolonged postoperative monitoring due to seromas (21, 22). Although postoperative pain scores were higher in the IPOM PLUS group, these patients experienced shorter hospital stays. This apparent discrepancy is explained by differences in postoperative care requirements rather than pain severity. IPOM PLUS patients did not require routine drains and had a lower incidence of seroma, allowing early discharge with oral analgesia. In contrast, SCOM/SCOLA involves creation of a subcutaneous space prone to fluid accumulation, and routine drain placement necessitated inpatient monitoring of output and delayed discharge. Therefore, duration of hospitalization in this cohort was primarily driven by postoperative management protocols rather than pain intensity.

Yanari et al. focused on hybrid IPOM PLUS as a solution for high-risk patients, including those with BMI values as high as 53.8 kg/m², whereas our study maintained a mean BMI of 26.95 across both groups, demonstrating the versatility of the technique in more standard patient populations. Additionally, our study highlighted shorter hospital stays with IPOM PLUS (mean of 1.83 ± 0.79 days) vs. SCOM/SCOLA (5.07 ± 1.36 days), reinforcing the efficiency benefits of IPOM PLUS in both routine and high-risk cases, as described by Yanari et al. for complex scenarios requiring extended care. Both studies affirm the adaptability and reduced recurrence potential of IPOM PLUS, but our findings emphasize that, even in routine cases, this technique offers significant advantages over alternatives (6).

Our findings underscore a critical balance in laparoscopic ventral hernia repair. IPOM PLUS appears advantageous in settings prioritizing surgical efficiency, shorter hospitalization, and reduced seroma risk, though with greater immediate discomfort and expense. Mechanistically, the higher pain scores in IPOM PLUS likely stem from intraperitoneal fixation with tackers or trans fascial sutures, whereas SCOM/SCOLA avoids peritoneal contact and thus reduces nociceptive stimulation. The subcutaneous plane, however, is prone to dead space and fluid accumulation, explaining the higher seroma rate. Importantly, most seromas were clinically mild and resolved with conservative management, indicating limited clinical impact despite higher incidence.

Although Intraperitoneal Onlay Mesh Repair has demonstrated favourable outcomes with respect to seroma formation and recurrence, Subcutaneous Onlay Laparoscopic Approach/Subcutaneous Onlay Mesh Repair continues to be utilized in many healthcare systems with limited resources. In such settings, cost considerations, availability of composite meshes, and access to advanced techniques such as enhanced-view totally extraperitoneal repair (eTEP) or robotic surgery may be constrained. Consequently, procedural affordability and technical feasibility remain important determinants of the surgical approach.

The objective of the present study was not to establish equivalence or superiority of SCOM/SCOLA over IPOM, but rather to provide a pragmatic comparison of short-term clinical outcomes and cost parameters between two minimally invasive techniques currently used in routine clinical practice. This perspective positions SCOM/SCOLA as a context-specific and selective alternative, rather than a replacement for IPOM.

From a clinical perspective, IPOM PLUS is well suited for patients requiring rapid recovery and minimal hospital stay, especially in high-volume or resource-optimized healthcare systems. Conversely, SCOM/SCOLA may be preferred for patients with low pain tolerance, cosmetic concerns, or when avoidance of intraperitoneal mesh is prioritized. Importantly, cost analysis showed that while IPOM PLUS incurs higher procedural expenses, its shorter hospital stay may offset long-term costs, a finding echoed by Parker et al. in a systematic review of economic analyses in hernia surgery (23).

Future research should focus on multicentre randomized controlled trials with larger sample sizes to validate these findings and provide greater external validity. Long-term follow-up is essential to assess recurrence, chronic pain, mesh integration, and quality of life. Formal cost-effectiveness studies incorporating patient-reported outcomes, such as EQ-5D or SF-36, are required to provide more meaningful economic comparisons. Additionally, strategies to mitigate seroma formation in SCOM/SCOLA, such as adjunctive drains, quilting sutures, or use of sealants, warrant investigation. Similarly, innovations in mesh technology, including partially absorbable or barrier-coated meshes, could help reduce both pain and seroma-related complications. Comparative studies involving newer approaches, such as robotic TAPP or robotic preperitoneal repairs, may also shed light on the evolving role of minimally invasive hernia surgery in optimizing patient outcome.

## Conclusion

This study highlights the distinct advantages and limitations of IPOM PLUS and SCOM/SCOLA techniques for hernia repair. While IPOM PLUS offers efficiency with shorter surgical times and hospital stays, SCOM/SCOLA provides better postoperative pain relief and cosmetic results. The longer hospital stay observed in the SCOM/SCOLA group was primarily related to postoperative drain management protocols rather than the surgical technique itself.

Both techniques have their place in clinical practice, and the choice of method should be tailored to individual patient needs and healthcare system priorities. These findings contribute to the growing body of evidence on hernia repair techniques and offer valuable insights for surgeons and healthcare providers in optimizing patient care. Overall, the choice between the two techniques depends on patient-specific factors. For patients prioritizing rapid recovery and minimal hospital time, IPOM PLUS is the preferred approach. On the other hand, SCOM/SCOLA is ideal for patients who benefit from lower postoperative pain and cost- effectiveness, albeit with a higher risk of seroma.

## Data Availability

The original contributions presented in the study are included in the article/Supplementary Material, further inquiries can be directed to the corresponding author/s.
